# Bipartite viral RNA genome heterodimerization influences genome packaging and virion thermostability

**DOI:** 10.1128/jvi.01820-23

**Published:** 2024-02-08

**Authors:** Yiyang Zhou, Andrew L. Routh

**Affiliations:** 1 Department of Microbiology and Immunology, The University of Texas Medical Branch, Galveston, Texas, USA; 2 Department of Biochemistry and Molecular Biology, The University of Texas Medical Branch, Galveston, Texas, USA; 3 Department of Immunology and Microbiology, Scripps Research, La Jolla, California, USA; 4 Sealy Center for Structural Biology and Molecular Biophysics, The University of Texas Medical Branch, Galveston, Texas, USA; 5 Institute for Human Infections and Immunity, University of Texas Medical Branch, Galveston, Texas, USA; Loyola University Chicago - Health Sciences Campus, Hines, Illinois, USA

**Keywords:** next-generation sequencing, Flock House virus, RNA-RNA interaction, virus genome packaging, thermostability, XL-ClickSeq

## Abstract

Multi-segmented viruses often multimerize their genomic segments to ensure efficient and stoichiometric packaging of the correct genetic cargo. In the bipartite Nodaviridae family, genome heterodimerization is also observed and conserved among different species. However, the nucleotide composition and biological function for this heterodimer remain unclear. Using Flock House virus as a model system, we developed a next-generation sequencing approach (“XL-ClickSeq”) to probe heterodimer site sequences. We identified an intermolecular base-pairing site which contributed to heterodimerization in both wild-type and defective virus particles. Mutagenic disruption of this heterodimer site exhibited significant deficiencies in genome packaging and encapsidation specificity to viral genomic RNAs. Furthermore, the disruption of this intermolecular interaction directly impacts the thermostability of the mature virions. These results demonstrate that the intermolecular RNA-RNA interactions within the encapsidated genome of an RNA virus have an important role on virus particle integrity and thus may impact its transmission to a new host.

**IMPORTANCE:**

Flock House virus is a member of Nodaviridae family of viruses, which provides a well-studied model virus for non-enveloped RNA virus assembly, cell entry, and replication. The Flock House virus genome consists of two separate RNA molecules, which can form a heterodimer upon heating of virus particles. Although similar RNA dimerization is utilized by other viruses (such as retroviruses) as a packaging mechanism and is conserved among Nodaviruses, the role of heterodimerization in the Nodavirus replication cycle is unclear. In this research, we identified the RNA sequences contributing to Flock House virus genome heterodimerization and discovered that such RNA-RNA interaction plays an essential role in virus packaging efficiency and particle integrity. This provides significant insight into how the interaction of packaged viral RNA may have a broader impact on the structural and functional properties of virus particles.

## INTRODUCTION

The packaged genome of RNA viruses encompasses a myriad of both short- and long-range RNA-RNA interactions. These RNA-RNA interactions mediate a variety of basic functions in the virus lifecycle, including replication, gene expression, and genome packaging (1–6). An important example includes the non-covalent association of two or more RNA genomic segments in multipartite virus families that are co-packaged into single virus particles. Retroviruses represent the most well-recognized case, in which two copies of the +ssRNA genome are homo-dimerized and encapsidated in virions (7, 8). In HIV-1, the dimerizing sequences and their protein partners have been well characterized (9). HIV-1 genomic dimerization is crucial for viral assembly and virion maturation, and indispensable for genome reverse transcription (RT) and recombination (7, 8). In a similar fashion, hetero-dimerization can also be presented in the form of intersegmental RNA-RNA interactions that occur between genomic RNA segments of multi-partite viruses such as bluetongue virus, rotavirus (2, 10–12), and influenza viruses (1, 13, 14). These transient intermolecular RNA-RNA interactions are required for *trans*-activation of RNA replication, segment assortment, and genome packaging (11–13).

One interesting case of viral genome heterodimerization is presented by Nodaviridae: a family of +ssRNA viruses with a small bipartite genome encapsidated into non-enveloped icosahedral particles. Both Flock House virus (FHV) and Nodamura virus, despite their serological and structural differences (15), heterodimerize their bipartite RNA genomes into a single RNA complex upon heating the virions (10). The *in vitro* heating of virion may provide the necessary energies to expediate certain *in vivo* conformational changes of virus, as demonstrated in picornaviruses (16). Once formed, the non-covalent heterodimerized viral RNA can withstand denaturing conditions during RNA extraction, as well as native gel electrophoresis (10). It is unknown whether such a “semi-permanent” genome heterodimerization plays a role in virus life cycles and whether such duplexing is required for the correct encapsidation of each RNA molecule. It is also unknown what sequences contribute to the formation of the heterodimer.

In this study, we use FHV as a model system to reveal the sequences related to heterodimer formation and to demonstrate how such intermolecular RNA-RNA interaction has important roles in multiple stages of the viral lifecycle. FHV is a versatile model system for the study of the structure and molecular biology of non-enveloped viruses. The small bipartite genome encodes the RNA-dependent RNA polymerase (RdRp) on RNA1 (3.1 kb) and the structural capsid protein on RNA2 (1.4 kb) (17). RNA1 also encodes a subgenomic RNA (RNA3) whose expression is regulated via a series of *cis*-acting elements (18) and yields protein B2 as an essential suppressor of cellular anti-viral silencing mechanisms (19). The replication of the FHV genome takes place in spherules formed by invaginations of outer mitochondrial membranes (20), where RNA1 replicates independently and *trans*-activates RNA2 replication (18, 21). Structurally, the *T* = 3 icosahedral FHV virion packages specifically one molecule of RNA1 and RNA2, whereas the subgenomic RNA3 is excluded (22, 23). Cryo-EM and X-ray crystallography studies revealed the encapsidated FHV genome forms a highly ordered dodecahedral cage of RNA (24, 25). It also has been demonstrated that the FHV RNA dodecahedral cage extensively interacts with the capsid shell with a clear structural tropism (26).

Virus-like particles (VLPs) of FHV can readily be expressed and generated in cell culture. Interestingly, VLPs predominantly package host RNAs in place of the viral genome (27–29). In spite of this, the molecular structure of VLPs is indistinguishable from that of authentic FHV virions containing the viral genome, and a dodecahedral cage of RNA is also observed. These findings indicate that the structure of mature FHV itself does not prevent the encapsidation of non-viral RNAs, so the virus must employ other strategies to ensure the encapsidation of viral genome. A stem-loop structure on RNA2 has been found to be essential for FHV genome packaging (30). Additionally, several capsid motifs have been shown to impact RNA recognition (31–33). Recent studies revealed FHV genome packaging may employ multiple synergetic packaging sites (26). Despite detailed characterization of the structure of mature FHV particles and the encapsidated genome, the molecular mechanisms that direct the specific and stoichiometric packaging of FHV RNAs still remain elusive.

In this study, we hypothesized that viral genome heterodimerization is formed as a result of RNA1-RNA2 interactions and that these specific intermolecular interactions impact both the efficiency and the specification of genome packaging of the virus. We demonstrate that the heterodimerized RNA can be crosslinked with a psoralen derivative: 4′-aminomethyl trioxsalen hydrochloride (AMT). This suggests the heterodimer is formed as a result of RNA1-RNA2 base pairing. We also introduce a novel next-generation sequencing (NGS) method: “*XL-ClickSeq”* (crosslink-ClickSeq), which is designed to specifically probe double-stranded RNA regions. We found that defective FHV particles retained heterodimer formation, in spite of containing large deletions in each genomic RNA, supporting a biological role of heterodimer. Thermodynamic prediction revealed one RNA1-RNA2 interaction site (RNA1:1092–1104 and RNA2: 277–288) that contributes to heterodimer formation. Recombinant viruses with disrupted heterodimer base pairing at this site exhibited significant reduction in packaging efficiency, without compromising the yield of capsid protein or genomic RNAs. Mutant viruses also displayed reduced packaging specificity, which encapsidated a reduced amount of RNA2 and more complement host RNAs than that of wild type (wt). We also developed a fluorescence-based method to monitor the thermostability of viral particles. Viruses with disrupted heterodimer base pairs showed significant reduction in thermostability in both molecular and high-throughput assays. These results support an important role for the FHV genome heterodimer in the virus lifecycle, particularly in governing the virus packaging process and structural integrity. The methods and techniques presented in this study shed light on the connection between RNA structure and the virus replication cycles. Importantly, beyond elucidating mechanisms for genome packaging, our findings demonstrate a fundamental and causal link between the higher-order structure of encapsidated genomic RNAs and the structural integrity of virus particles. Therefore, rather than being a passive cargo, the viral genomic nucleic acid itself plays an important role in virus particle integrity and stability and thus its transmission to a new host.

## MATERIALS AND METHODS

### Cell culture and virus

Wild-type Flock House virus was generated from transfecting *Drosophila melanogaster* (S2) cells. The detailed protocol regarding transfection, S2 culture maintenance, and virus passaging was described previously (26). In most of this study, wt virus and mutants were purified with standard methods (26), which sequentially consist of 4% polyethylene glycol (PEG) 8000 precipitation, RNase/DNase digestion, sucrose gradient ultracentrifugation, and 100K MWCO polyethersulfone (PES) membrane filtration. During packaging specificity and thermostability experiments, quintuple-purified virus stocks were used to ensure minimum non-viral content: after standard purification, the purified viruses were digested again with RNase/DNase and purified again with sucrose gradient and PES membrane filtration. In total, this quintuple purification procedure encompasses 1× PEG precipitation, 2× sucrose gradient purification, 2× PES membrane filtration, and 2× nuclease treatment.

### Heterodimer formation

Purified FHV virions (in 50 mM HEPES, pH 7.2) were heated to 65°C for 10 min in a thermocycler. This was followed by slow cooling at a pace of −1°C per 5 s until 4°C. A number of RNA extraction methods were experimented (see Discussion). In this study, all heterodimer RNAs were extracted and retained with Quick-RNA Viral Kit (Zymo Research) with standard manufacturer’s protocol. The extracted heterodimer was kept at 4°C until further analyses. The electrophoresis of heterodimer RNA was conducted under non-denaturing conditions, with 1% agarose, 1× lithium acetate borate buffer, and a custom loading dye consisting of 2.5% Ficoll-400, 6.6 mM Tris-HCl ph 8.0, and 0.01% OrangeG.

### Northern blot

The heterodimer bands were cut from agarose gel (Fig. 4a), and the RNA content was recovered with Zymoclean Gel RNA Recovery Kit (Zymo Research) using standard procedures. Equal amounts of RNA were heat denatured (65°C 10 min). Two fluorescently labeled ssDNA probes were designed to specifically target the conserved FHV +RNA1/+RNA3 and +RNA2 sequences, respectively (RNA1/RNA3: Cy5-GAGTGTTGGTTTTGCCTCCT; RNA2: Cy3-GAAACGCCAAACCAGGTTGACTTAATCTGGTTAGCGCCGCCATGTTCAT). Electrophoresis, membrane transfer, and hybridization were conducted with NorthernMax (Ambion) Kit and protocol with modifications: (i) during electrophoresis, a dye-less loading buffer was made to prevent fluorescent interference; (ii) transferred nylon membrane was UV crosslinked with UV stratalinker 2400 (1,200 µJoules); (iii) hybridization was conducted with ULTRAhyb-Oligo buffer instead of ULTRAhyb at 42°C overnight, with 4 µL of each probe at 10 mM; (iv) post-hybridization, membrane was sequentially washed twice with low stringency wash (5 min RT), once with low stringency wash (2 min 42°C), twice with tris-buffered saline and 0.1% Tween-20 buffer wash (5 min RT), and twice with tris-buffered saline buffer (5 min RT); (v) membrane was exposed on Typhoon (FLA9500) to detect fluorescence. Densitometry was conducted with ImageJ (NIH). Total cellular RNAs and supernatant RNAs (Fig. 5e through g) were extracted with Direct-zol RNA Kit (Zymo Research), and northern blot assays were conducted as before. Statistical assays were conducted with one-tailed paired *t*-test for means, with α = 0.05, *N* ≥3 biological replicates.

### Oxford nanopore technology MinION sequencing

The RNA extracted from defective FHV particles was sequenced with ONT MinION. RNA was reverse transcribed, PCR amplified, and ligated with KIT SQK-LSK109 with standard methods and sequenced with a MinION R9.4 flowcell. The detailed library protocol, software settings, data processing, and genome alignment methods are previously described (34).

### AMT crosslinking

FHV RNAs extracted from heated particles were crosslinked with 4′-aminomethyl trioxsalen hydrochloride (AMT). An amount of 1 µg of purified RNA is mixed with AMT (at 20 µg/mL) and placed 5 cm underneath a 365-nm UV light (3UV-38, UVP) for 38 min on ice in a dark room. This yielded approximately 0.15 J/cm^2^ of energy. The crosslinked RNA was purified with RNA Clean & Concentrator (Zymo Research) to remove excessive AMT.

### XL-ClickSeq

AMT-crosslinked RNA was directly used as template during reverse transcription. Standard ClickSeq protocol (35) was carried out. In brief, 250 ng of templated RNA was supplemented with a mixture of azido-NTP:dNTP (1:5 molecular ratio) during random primed reverse transcription. This is followed by “Click-ligation” (36) with a 5′-hexynyl-functionalized Illumina adapter (IDT). The adapter ligated cDNA fragments were subsequently PCR amplified to complete Illumina adapter sequences and to incorporate sample indexes/barcodes. The ClickSeq libraries of non-crosslinked FHV RNAs were constructed the same way as for the control.

### Bioinformatics

The bioinformatics of XL-ClickSeq followed a published bioinformatic pipeline (26). In brief: the raw Illumina sequencing data were sequentially prepared by trimming Illumina adapter [*cutadapt* (37): -b AGATCGGAAGAGC -m 40], trimming of nucleotides adjacent to the triazole linkage in the “click-linked” cDNA using *FASTX toolkit* [http://hannonlab.cshl.edu/fastx_toolkit/: (fastx trimmer -Q33 -f 7)], and filtering read quality (fastq_quality_filter -Q33 -q 20-p 96). End-to-end alignment to the FHV genome (NC 004146 and NC 004144 for RNA1 and RNA2, respectively) was conducted [*Bowtie* (38): -v 2 --best]. The genome coverage data were extracted from the aligned reads [SAMtools (39): view/sort/mpileup], and the genome coverage of crosslinked heterodimer was compared with non-crosslinked control RNA.

For both DMS-MaPseq and HD mutant-packaged RNA characterization, the generated azido-tagged cDNAs were “click” ligated with an Illumina adapter with 12 random nucleotides to act as unique molecular identifiers for de-duplication and control of PCR bias. Raw reads were processed to remove Illumina adapter and to assign unique identifier [*fastp* (40): -a AGATCGGAAGAGC -U --umi_loc read1 --umi_len 14 --umi_prefix umi -l 30]. For DMS-MaPseq, *Bowtie2* (41) was used to align reads to FHV genome to allow gapped alignment (--local). This is followed by de-duplication to remove PCR bias [*umi-tools* (42): dedup --method=unique] and extraction of mutation rate with a minimum nucleotide coverage of 1,000 [SAMtools (39): view/sort/mpileup]. For packaged RNAs of mutant viruses, *Hisat2* (43) was used for alignment with default settings to FHV genome first, while the unmapped reads were further aligned to *Drosophila melanogaster* genome [release 6 (44), GCA_000001215.4]. This was followed by de-duplication and read extraction as stated above. To characterize the packaged RNAs of viruses, statistical assays were conducted with one-tailed Student *t*-test (equal variance), with α = 0.05, *N* ≥3 biological replicates.

### RNA structure thermodynamic prediction

Free energy-based thermodynamic prediction was conducted with *RNAstructure Web Server* (45), with “bifold” algorithm to allow both inter- and intramolecular base pairs (30 nts. maximum loop size with 5% maximum energy difference at 310.15K). Six RNA1 heterodimer candidate sites (whose flanking sequences grouped into five candidate regions) and six RNA2 candidate sites (whose flanking sequences grouped into six candidate regions) were cross-matched for RNA secondary structure prediction (Fig. S3). The predicted structure file was re-organized, re-colored for graphical purposes with *StructureEditor*, which is provided by *RNAstructure* suite.

### Mutant virus cloning, transfection, and passaging

The primers used to generate the In-Fusion cloning (TaKaRa) fragments were listed in Table S1. Universal upstream (TGCATAATTCTCTTACTGTCATGCCATCCGTAAG) and downstream (TAAGAGAATTATGCAGTGCTGCCATAACCATG) primers were used to target the backbone of pMT plasmids (Invitrogen). The primers used to generate additional clones (Fig. S8) were listed in Table S2, with the same universal primers provided above. Overlapped PCR fragments were generated (Phusion High-Fidelity DNA Polymerase, NEB) and cloned into competent cells with standard In-Fusion HD cloning techniques. The plasmids containing mutated viral sequences were sanger sequenced to confirm the mutation(s). Equimolar RNA1 and RNA2 plasmids containing mutated heterodimer sequences were used to co-transfect S2 cells with Lipofectamine 3000 (Invitrogen) with standard protocols. 500 µM of copper sulfate was added to the culture 24 h post transfection to induce the pMT promoter, of which viral sequences were placed behind. The generated P0 viruses were serially passaged up to P2 with 3 days interval.

### DMS-MaPseq

Dimethyl sulfate (DMS) RNA methylation method was described previously (26). In brief, DMS was used at 5% final concentration with purified viruses for 5 min at 30°C. This was followed by 5 min quenching with excess volume of 10 mM Tris pH 7.4 and 30% 2-mercaptoethanol (BME). RNA was extracted from DMS-treated virions, and RNAseq libraries were constructed using ClickSeq as previously stated (26). For each virus (wt/HD1/HD2), the nucleotide mutation rate of DMS-treated virus was compared with that of the untreated and corresponding control virus to generate signal. To distinguish DMS-MaPseq signals from background, a background threshold was applied as previously described (26), which is determined as the highest 5% mutation rate of the corresponding control.

### Relative cytotoxicity

In this study, the relative cytotoxicity of each virus was measured by alamarBlue cell viability assay of P0-transfected S2 cells, as described previously (26). In brief, 25k naïve S2 cells were seeded in black 96-well plate, transfected with 100 ng plasmids of wt or mutant virus genome, and induced with copper sulfate. Then, 2 days post induction, alamarBlue cell viability reagent (ThermoFisher) was supplemented. Cells were incubated for 4 hours, before fluorescence detection with an EnSpire plate reader (PerkinElmer) at 560 nm excitation and 590 nm emission. The relative fluorescence, which indicates viable cell count, was then normalized reverse ratiometrically to relative cytotoxicity, with mock transfection = 0% and FHV wt transfection = 100%. Statistical assays were conducted with one-tailed Student *t*-test (equal variances), with α = 0.05, *N* ≥6 biological replicates.

### Western blot

Three days post transfection, equal volume of transfected S2 cells (P0 virus) was used to inoculate naïve cells to generate P1 viruses. And, 24 h post inoculation, 100 µL culture was collected. Cell and supernatant fractions were separated with centrifugation (1,000 × *g*, 10 min, 4°C). Cell fraction was washed twice and resuspended into 30 µL with 1× phosphate-buffered saline and 1× cOmplete proteinase inhibitor (Roche). Supernatant fraction was supplemented with 1× cOmplete and reduced to 30 µL with vacuum centrifuge. Entire 30 µL of both cellular and supernatant fractions was loaded on to a Bolt 4%–12% Bis-Tris Plus gel (Invitrogen). Details regarding membrane transfer and western blotting are documented previously (26). In brief, a rabbit anti-FHV polyclonal antibody and an Alexa Fluor 488 goat anti-rabbit IgG (Invitrogen) were used to probe FHV alpha, beta, and gamma peptides. Prior to membrane transfer, part of SDS-PAGE gel was cut and stained (Coomassie brilliant blue R-250) to highlight α-tubulin (55 kDa) as a loading control. Labeled membrane was visualized with Typhon (FLA9500), and densitometry was conducted with ImageJ (NIH). Statistical assays were conducted with one-tailed Student *t*-test (equal variances), with α = 0.05, *N* ≥4 biological replicates.

### High-throughput thermostability assay

A high-throughput virion thermostability assay was developed by repurposing a qRT-PCR program. Since intact FHV is impermeable to fluorescent nucleic acid stain, it is feasible to measure the fluorescence increase of stain/virus mixture, as an indication of permeability change (virion melting) in relation to temperature. In this assay, it is important to ensure the purity of virions and the minimum of co-purified, non-encapsidated cellular RNA. As detailed above, all viruses were quintuple purified which consists of multiple purification as well as two rounds of RNase A/DNase I digestion. A 50-µL mixture consisting of 50 µg of purified particle, 50 mM HEPES (pH 7.2), 1× nucleic acid stain (GelStar, Lonza) was added to a 96-well qRT-PCR plate. A thermocycling program was set on a qRT-PCR machine (Roche LightCycler 480II) (Fig. 6b), which comprised a 1-min incubation cycle with gradually increased temperature (1°C per cycle, from 37°C to 95°C) and a 10-s cooling at 37°C. Fluorescence reading was taken at the end of each cooling step to avoid any temperature-induced absorbance change of nucleic acid dye. The observed temperature-to-fluorescence curve of different particles can be transformed to a fluorescence-to-temperature formula, which is generated by converting the polynomial (order = 2) trendline of average fluorescence change (from 47°C to 62°C). The melting temperature of each virus particle (Tm, which is defined as the temperature to reach 50% fluorescence increase) can be calculated for each replicate thereafter. Statistical assays were conducted with one-tailed Student *t*-test (equal variances), with α = 0.05, *N* = 12 (each sample consisted of two biological replicates, each biological replicate consisted of six technical repeats).

## RESULTS

### FHV heterodimer formation could be thermostabilized by psoralen crosslinking

**Fig 1:**
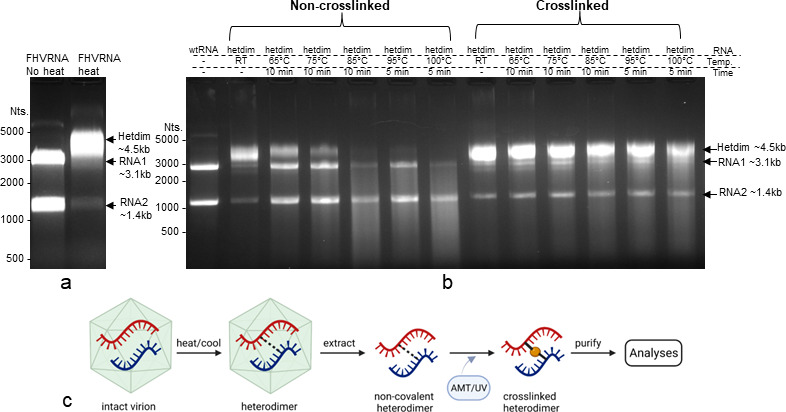
FHV RNA1-RNA2 base pairing contributed to heterodimer formation. (**A**) Upon heating of virions, the majority of FHV RNA1 and RNA2 formed a heterodimerized RNA duplex (*hetdim*), shown as a band ~4.5 kb on non-denaturing agarose gel. (**B**) Heterodimerized FHV RNA was subjected to 4′-aminomethyltrioxsalen hydrochloride (psoralen) and UVA light to induce cross-strand pyrimidine crosslinking. The crosslinked heterodimer band can withstand heat up to 100°C for 5 min, while the non-crosslinked heterodimer bands gradually denatured into separate RNAs with temperature increases. And, 500 ng of total RNA was used in each lane. The molecular weight markers are referenced with ssRNA ladder, which only represent the approximate RNA size estimation under non-denaturing condition. (**C**) Schematic representation of the method for crosslinking FHV-heterodimerized RNAs after extraction.

The stable heterodimerization of each of the two genomic segments of Flock House virus to form an RNA1-RNA2 duplex upon heating and cooling of FHV particles has previously been well described (10). Interestingly, the formation of this heterodimer only occurred within the context of authentic virus particles and was not formed *in vitro* with purified viral RNAs alone (10). This suggests that the assembled virus particles impose one or more well-defined and conserved interaction points between each genomic segment, although the functional importance is not characterized. To address this and to identify the potential RNA1-RNA2 duplex interaction sites, we recreated the heterodimerization by heating purified FHV virions at 65°C for 10 min, followed by slow cooling at a rate of −1°C/5 s until 4°C and RNA extraction. Upon electrophoresis on non-denaturing agarose gel, the RNA1-RNA2 heterodimer (*hetdim*) appeared as the predominant RNA species, with a small amount of monomeric RNA1 and RNA2 (Fig. 1a). The broad molecular weight range of the heterodimer band (3.5k–5k) indicates the heterodimer may consist of multiple RNA conformations.

Psoralens are planar photochemical reagents that can intercalate double-stranded nucleic acids and form inter-strand covalent crosslinks between adjacent pyrimidines upon UVA irradiation (46). To investigate whether FHV RNA heterodimer is formed as a result of RNA1-RNA2 base pairing, a psoralen derivative 4′-aminomethyl trioxsalen hydrochloride (AMT) was supplemented to extracted RNAs from heated virions, which consisted mainly of heterodimerized viral RNA (Fig. 1a). After crosslinking with 365 nm UVA light, the AMT-crosslinked heterodimer became resistant to heat denaturation at temperatures up to 100°C (Fig. 1b). In comparison, non-crosslinked heterodimer began to denature at 65°C and were completely monomerized at 85°C. This demonstrates that the heterodimerization is a result of inter-strand base pairing between RNA1 and RNA2.

### “*XL-ClickSeq”* to identify dsRNA interaction sites

**Fig 2:**
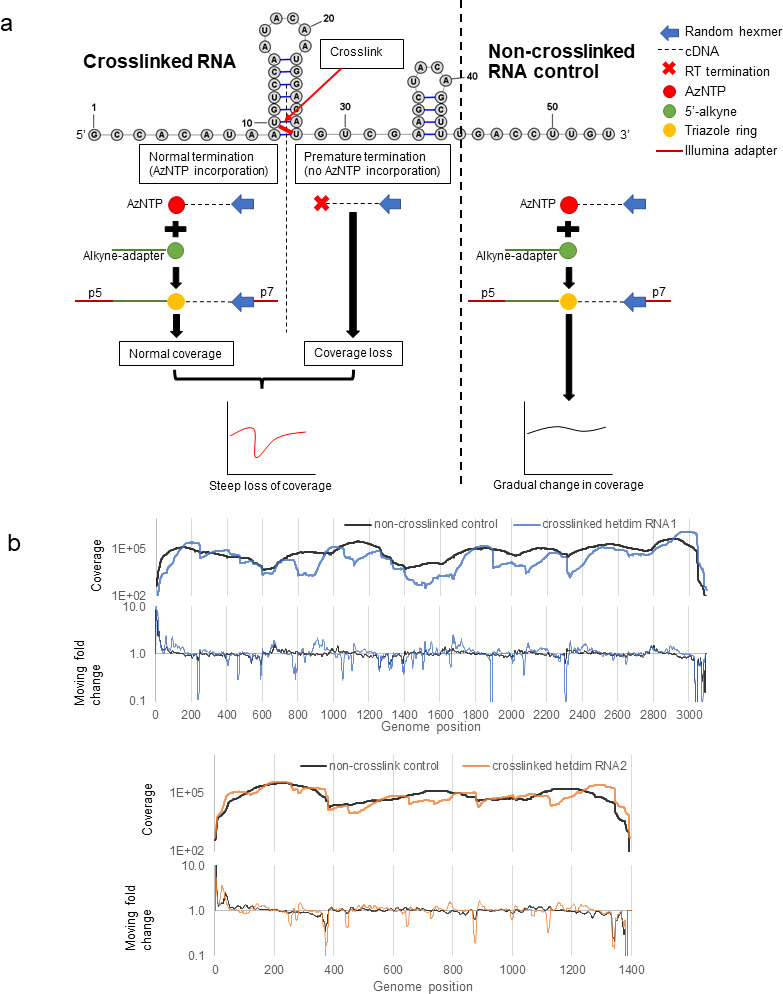
“XL-ClickSeq” revealed heterodimer candidate sites. (**a**) “XL-ClickSeq” method combines psoralen crosslinking (“XL”) with “ClickSeq” to specifically probe crosslinked double-stranded RNA regions. RNA molecule is UV crosslinked with psoralens (AMT). The cross-stranded covalent bonds prevent AzNTP incorporation during RT, which subsequently impedes the click ligation to an alkyne-functionalized Illumina adapter. The RNA base pairing is thereafter revealed by the sharp coverage loss near crosslinking sites. (**b**) XL-ClickSeq of heterodimerized FHV RNA revealed several sites with sudden loss of coverage, indicating double-stranded RNA duplexes. Coverage data of crosslinked heterodimer were normalized and averaged (*N* = 3).

Having confirmed that AMT crosslinking preserves the interaction between RNA1 and RNA2 in the FHV heterodimer, we employed next-generation sequencing approaches to identify the interaction site(s). We developed a novel approach that we call “*XL-ClickSeq,*” which combines psoralen crosslinking (“XL”) and “ClickSeq” (47) to reveal the potential sites of AMT-crosslinked double-stranded structures of RNA (Fig. 2a). Random-primed ClickSeq relies on the stochastic incorporation of 3′-azido-deoxynucleotides (AzNTPs) to randomly terminate cDNA synthesis during RT of unfragmented RNA templates and subsequently “click-ligate” (36) a 5′-alkyne-functionalized oligonucleotide containing an Illumina sequencing adaptor. In XL-ClickSeq, unfragmented RNA template is first AMT crosslinked. The AMT crosslink induced cross-strand covalent bonds which prevent the elongation of the nascent cDNA past the crosslink site and therefore prevents the required incorporation of an AzNTP near this site. This prevents click ligation to the click adapter and thus prevents PCR amplification and subsequent sequencing on an Illumina flowcell. This results in a loss of sequence read coverage at the 3′ of crosslinking sites. By contrasting to the normal coverage of non-crosslinked genomic regions, steep drops in genomic coverage indicate crosslinked dsRNA regions at near-nucleotide resolution.

Heterodimerized FHV RNAs were extracted from heated virions and crosslinked with AMT (Fig. 1b). XL-ClickSeq of crosslinked heterodimer revealed that numerous genomic locations showed apparent loss of coverage, which was distinctively absent from non-crosslinked control RNAs (Fig. 2b). This is further highlighted by moving fold change data (10 nt. interval, Fig. 2b). Sharp changes in coverage are characteristic of XL-ClickSeq and indicative of the locations of crosslinked dsRNA site. With the XL-ClickSeq data of the heterodimer, we applied a manual threshold to distinguish dsRNA sites from background: a candidate site needs to contain consecutive (greater than 6) nucleotides that have a moving fold change reduction (10 nt. interval) greater than 25% (Fig. S1). We also excluded the sites located within the 3′-most 100 nts of both RNAs since reduced genomic coverage at 3′ termini is common in RNAseq (47), and such loss of coverage was seen on both crosslinked RNA or non-crosslinked control. We found a total of 18 crosslinking sites on RNA1 and 9 on RNA2, respectively. It is important to note that the revealed dsRNA sites do not differentiate between intramolecular and intermolecular base pairing.

### FHV heterodimer formation was conserved in defective particles

We have previously characterized the emergence and evolution of defective viral genomes of FHV during serial passaging in cell culture (34). The “mature” defective RNAs (D-RNAs) of FHV are characterized by large deletions of genomic RNA but retain important functional RNA motifs such as those required for RNA replication and encapsidation. To determine whether the RNA1-RNA2 intermolecular interactions are also conserved in “mature” D-RNAs, we investigated whether FHV heterodimer can be formed in defective particles (assembled virions that encapsidate D-RNAs). Stocks of defective FHV particles were obtained from the supernatant from *Drosophila* S2 cells in culture after seven serial rounds of passaging (P7). Nanopore sequencing revealed that P7 FHV virus stock consisted of 92% D-RNA1 and 88% D-RNA2 (Fig. S2). These D-RNAs are characterized by four predominant genomic deletions (nt. 318–940 and nt. 1261–2265 on RNA1, nt. 251–512 and nt. 717–1217 on RNA2) (Fig. S2). In comparison, early passage of FHV (e.g., P2) comprised less than 7% of defective RNA1 and 4% defective RNA2 (34), and hence, considered wild-type genome in this study.

**Fig 3:**
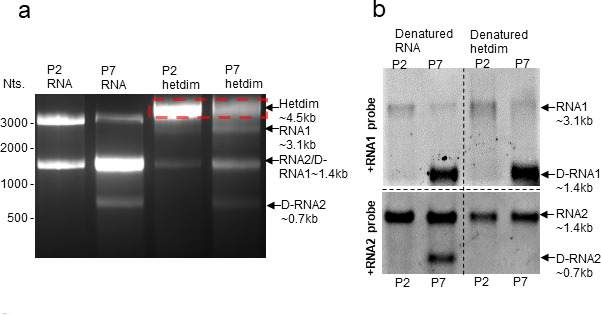
FHV RNA heterodimerization is conserved in defective particles. (**A**) Both P2 and P7 FHV viruses can form heterodimer despite P7 virus comprising primarily defective FHV particles, in which RNA genomes contain large deletions (D-RNA1 and D-RNA2). The heterodimer bands (red box) were excised, and RNAs were recovered and denatured for subsequent northern blot assay. (**B**) Northern blots of non-heterodimerized RNA (denatured RNA) and heterodimerized RNA (denatured heterodimer) from P2 and P7 particles. The D-RNAs from P7 particles are clearly shown. Only D-RNA1 can be detected from P7 particle heterodimer band. The molecular weight marker is referenced with ssRNA ladder, which only represents the approximate RNA size estimation under non-denaturing condition.

Surprisingly, despite the truncated genome, the P7 defective FHV particles were still able to form heterodimer upon heating (Fig. 3a). On non-denaturing agarose gel, the predominant P7 heterodimer species migrated similarly to that of wild type (P2) at around 4.5 kb, although there could be uncharacterized P7 heterodimer species at lower molecular weight. This suggests that in defective FHV particles, the heterodimer may comprise two copies of D-RNA1 molecules since D-RNA1 is approximately half the molecular weight of full-length RNA1.

To confirm that the heterodimer indeed consists of both RNA1 and RNA2, the predominant heterodimer species of both wild-type (P2) and defective (P7) viruses were extracted from non-denaturing agarose gel (red box in Fig. 3a). The retained heterodimer RNAs were then denatured and analyzed by northern blot using probes against +ssRNA1 and +ssRNA2 (Fig. 3b). D-RNA1 (~1.4 kb) and D-RNA2 (~0.7 kb) were readily detectable in P7 D-FHV. Surprisingly, the P7 heterodimer consisted of full-length RNA1, full-length RNA2, D-RNA1, but not D-RNA2. The exclusion of D-RNA2 from P7 heterodimer suggests that the heterodimerization may be interfered by the deletions in D-RNA2 while unaffected by the deletions in D-RNA1.

### Potential heterodimer sites and disruptive mutants

The previous experiments provided a range of constraints on where in the FHV genome the RNA1-RNA2 duplex interaction sites must occur. The presence of FHV D-RNA1 and absence of D-RNA2 in P7 heterodimers (Fig. 3b) indicate that at least one heterodimer site in RNA1 resided in the conserved regions of D-RNA1 (RNA1 nts. 1–317, 941–1260, 2266–3107), while the other complementary site in RNA2 must reside in a region deleted in D-RNA2 (RNA2 nts. 251–512, 737–1217). Therefore, of the 27 crosslinking sites identified by “XL-ClickSeq,” we further refined our selection to six sites on RNA1 and six sites on RNA2 that were found in these regions, and hence, may contribute to the formation of RNA1-RNA2 heterodimer (Fig. S3).

**Fig 4:**
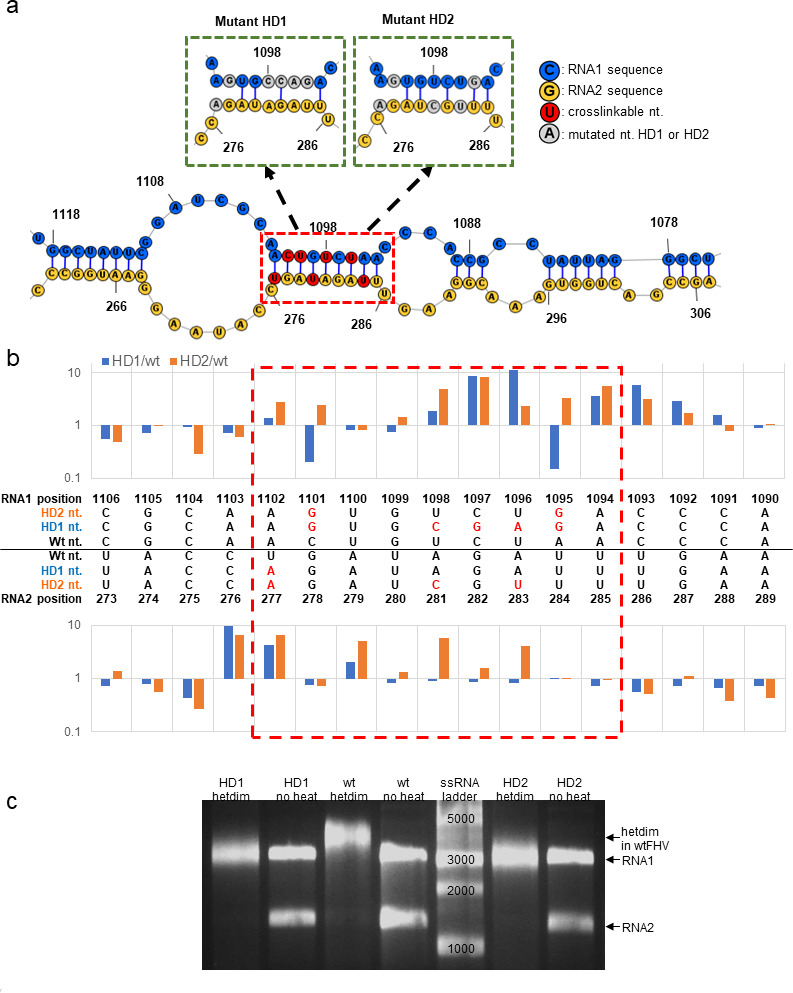
Potential heterodimer sites and disruptive mutants. (**a**) The predicted RNA1: 1093–1104 and RNA2: 276–286 base pairing (red box, based on cross-matching results from Fig. S3) contains several cross-strand pyrimidines available for AMT crosslinking (U-U and C-U). Two mutant viruses were created (HD1 and HD2), with synonymous mutations designed to disrupt the predicted base pairing in this region. (**b**) DMS-MaPseq of mutated viruses demonstrated more unpaired bases in the heterodimer stem (red box) as well as flanking sequences. Signals represent the fold change of mutation rate between mutant and wt viruses. (**C**) Upon heating, mutant (HD1 and HD2) viral RNAs can still be heterodimerized but with altered conformation, which migrated faster than heterodimer from wt virus in a native agarose gel.

In order to understand which sites can form intermolecular base-pairing duplexes, we used the “*bifold*” function of *RNAstructure* (45) to perform thermodynamic prediction of these candidate sites for both intermolecular and intramolecular base-pairing probabilities (Fig. S3). Each RNA1 candidate site as well as the flanking sequence (71–120 nts. in length) was cross-matched with each RNA2 candidate site and flanking sequence to investigate whether the selected candidate segments have greater potential to form intermolecular duplexes (RNA1-RNA2) than intramolecular ones (RNA1-RNA1 or RNA2-RNA2), whether the formed intermolecular base pairs are at the predicted crosslinking sites, and whether these base pairs exhibit crosslinkable pyrimidines (Fig. S3). One particular match (RNA1 nt. 1102 and RNA2 nt. 280) resulted in a 9-base-pair RNA duplex between RNA1: 1094–1102 and RNA2: 277–285, which is located at the proximity of a predicted crosslinking site (Fig. 4a). This double-stranded stem comprised four pairs of cross-stranded U-U and U-C pairs that are available for AMT crosslinking (46, 48). Beyond the nine base-pairing RNA stem, RNA1-RNA2 duplexing is also predicted in adjacent sequences. Between RNA1: 1075–1118 and RNA2: 261–307, 66% of the bases are predicted to be involved in RNA1-RNA2 base pairing. We highlighted the locations of this predicted heterodimer stem in relation to FHV ORFs and other known functional RNA motifs (Fig. S11).

To scrutinize this potential dimerization site between RNA1:1094–1102 and RNA2:277–285, we designed two mutant viruses (HD1 and HD2) that introduce synonymous mutations to disrupt the predicted base pairing of this double-stranded intermolecular stem. HD1 contained 5 nts. substitutions on RNA1 and 1 nt. substitution on RNA2, to predictively disrupt 5 base pairs. HD2 contained 2 and 3 nts. substitutions on RNA1 and RNA2, respectively, to predictively disrupt 4 base pairs (Fig. 4a). After transfection, the mutant viruses were continuously passaged. Illumina sequencing confirmed that the mutations were preserved in passage 2 (P2) viruses (Fig. S4).

We first sought to confirm whether RNA secondary structure in the predicted heterodimer region was disrupted by the synonymous mutations in the HD1 and HD2 virus genomes. WT, HD1, and HD2 viruses were purified with polyethylene glycol precipitation, sucrose gradient, and molecular weight filtration [as previously described (26)] and then subjected to in-virion dimethyl sulfate mutational profiling with sequencing (DMS-MaPseq) (26, 49). DMS-MaPseq uses dimethyl sulfate to methylate unpaired adenines and cytosines [and guanine to a lesser extent (26, 50)]. Conventional DMS-seq captures the methylated nucleotides at the termini of the halted reverse-transcribed cDNAs. In comparison, DMS-MaPseq provides a high-throughput approach by using a specific reverse transcriptase to tolerate methylated bases and detecting the methylation-induced mutations to report base pairing. In this study, we compared the DMS-MaPseq signals between mutant and wild-type viruses at the predicted heterodimer dsRNA regions (9 bps, RNA1: 1094–1102; RNA2: 277–285, red box of Fig. 4b). Seven nucleotides in HD1 and 14 nucleotides in HD2 showed increased DMS signal (defined here as the nucleotide error rate) relative to that of wild-type virus. Furthermore, in both mutants, additional nucleotides also exhibited higher DMS signal at the 5′ proximal of the predicted heterodimer stem. This indicates the introduced mutations disrupted the base pairing of the 9-bps heterodimer stem as well as some flanking bases. It is possible that the disrupted RNA base pairs in these mutants underwent compensatory conformational changes to reform base pairing with other RNA regions. This may explain why HD1 had fewer bases with increased DMS reactivity than HD2 despite bearing more disruptive mutations.

The conformational change of HD1 and HD2 could also be observed when these mutant viruses were heated to extract heterodimer (Fig. 4c). Despite disruption of the identified heterodimer stem, both HD1 and HD2 still formed heterodimers. This indicates there are multiple heterodimerization sites beyond the predicted region. Importantly, the heterodimers of both HD1 and HD2 mutants migrated faster than that of wt virus on non-denaturing agarose gel (Fig. 4c). This indicates that the disrupted stem of HD1 and HD2 triggered significant RNA conformational changes, which led to the observed electrophoresis mobility shift. Previously, it has been demonstrated that local RNA substitutions can lead to significant reconfiguration of the genomic RNA structure (26, 51). The heterodimer bands of HD1 and HD2 were extracted, denatured, and northern blot analyzed to verify that they indeed consisted of both RNA1 and RNA2 (Fig. S5).

As stated above, it is conceivable that there are multiple interaction sites between RNA1 and RNA2, in addition to the discovered stem (RNA1: 1093–1104 and RNA2: 276–286). To confirm this, we designed a molecular approach using DNA-oligo-guided site-specific cleavage by RNase H (Fig. S6). We designed DNA probes to specific sites flanking RNA1: 1093–1104 and confirmed their site-specific cleavage of monomerized viral RNAs (Fig. S6). However, the heterodimerized viral RNA resisted perturbation, as both RNA1 and RNA2 still migrated as a single heterodimer band in the native agarose gel. This suggests that there are additional undiscovered RNA1-RNA2 interaction sites.

### Disrupted heterodimer impeded virion assembly and genome packaging specificity

**Fig 5:**
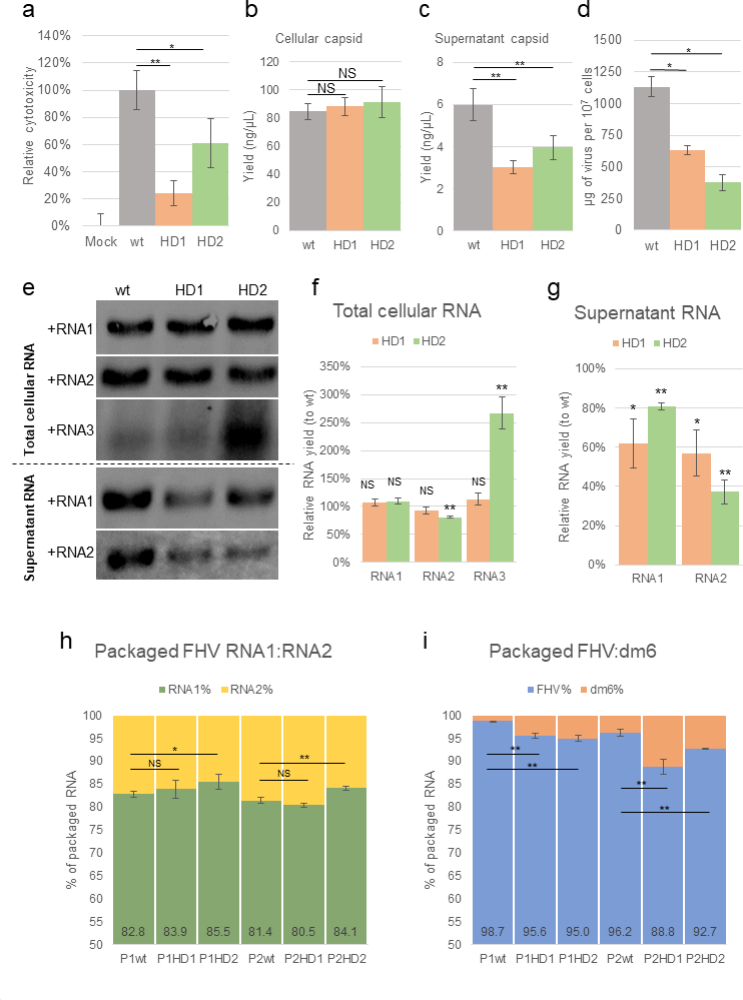
Disrupted heterodimer impeded virion assembly and packaging specificity. (**a**) alamarBlue assay revealed that both mutant viruses (HD1 and HD2) showed significantly reduced cytotoxicity than that of wild type (*N* ≥6 biological replicates). (**b**) Comparable amounts of capsid were detected by westernblot assays in cells infected by wt or mutant viruses (*N* ≥4 biological replicates). (**c**) Mutant viruses yielded significantly lesser capsid in supernatant (*N* ≥4 biological replicates). (**d**) Both mutant viruses also showed reduced yield in mature virions (*N* ≥3 biological replicates). (**e**) Northern blot of the infected cells or supernatant. (**f and g**) HD2-mutant virus triggered a significantly increased sgRNA3 in infected cells, which may be the cause of the slight but significant reduction of RNA2 (*N* ≥3 biological replicates). (**h**) HD2 mutant encapsidated significantly more RNA1 and lesser RNA2 than that of wild type in both P1 and P2 viruses (*N* ≥3 biological replicates). (**i**) Both HD1 and HD2 packaged significantly more host RNAs than that of wt (*N* ≥3 biological replicates). All statistical assays were conducted with one-tailed *t*-test for means (equal variances), with α = 0.05, NS, not significant; *, *P* < 0.05; **, *P* < 0.01.

We further sought to characterize HD1 and HD2 mutants to understand if the disrupted heterodimer site could impact virus lifecycle and fitness. alamarBlue assays were conducted to compare the cytotoxicity of mutant viruses to wild type. After transfecting equal amounts of plasmids containing sequences for wtFHV, HD1 and HD2, the cells transfected by HD1 and HD2 mutants (P0) showed significantly reduced cytotoxicity compared to that of wild type (Fig. 5a), indicating that both HD1 and HD2 might propagate slower than wt. Naïve cells were then inoculated with equal volume of transfected cell culture (P0). The resultant P1 culture was analyzed with western blot to investigate the capsid yield in cytosol and supernatant (Fig. 5b and c). Interestingly, neither mutant resulted in significantly different yields of capsid in cytosol (Fig. 5b). In contrast, both HD1 and HD2 yielded significantly reduced capsid in supernatant fraction (Fig. 5c; Fig. S7), where mature virus accumulates. The reduced supernatant capsid yields are consistent with reduced cytotoxicity of mutants (Fig. 5a). The reduced virion production of HD1 and HD2 was also confirmed by quantitating the yield of P2 virions after sucrose gradient purification, which was diminished for both the HD1 and HD2 mutants (Fig. 5d). Overall, this suggests that HD1 and HD2 mutations resulted in a deficiency in assembling mature virions that escaped infected cells despite HD1 and HD2 expressing comparable amounts of capsid proteins intracellularly to that of wt.

We next sought to determine whether the mutated heterodimer stem would impact viral replication and RNA packaging. Also, 1 µg of total cellular RNA or 300 ng of supernatant RNA was analyzed by northern blot with ssRNA probes specific for each viral RNA (Fig. 5e). Interestingly, HD2 accumulated significantly more sub-genomic RNA3 in cells and a slight but significant reduction of RNA2 (Fig. 5e and f). The negative yield correlation between RNA2 and subgenomic RNA3 is consistent with previous findings in FHV and other alphanodaviruses, which report that the replication of RNA2 not only is *trans*-activated by sgRNA3 but also suppresses the accumulation of sgRNA3 after being *trans*-activated (52–56). This suggests the disrupted heterodimer stem (RNA1:1094–1102 and RNA2: 227–285) may involve a novel function in the coordination of RNA2 and sgRNA3 replication. In supernatant fractions, both HD1 and HD2 showed significantly reduced RNA1 and RNA2 accumulation compared to that of wt (Fig. 5e through g), consistent with the lower yields of mature virions from that of mutant viruses.

We next investigated whether the disrupted heterodimer stem would impact virus genome packaging. P1 and P2 viruses of HD1, HD2, and wt viruses were collected and quintuple purified to ensure minimal unpackaged RNA content. The encapsidated RNAs were extracted from P1 and P2 viruses and characterized by random-primed RNAseq to determine the relative abundance of encapsidated virus and host RNAs (28, 29). We observed that HD2 packaged significantly more RNA1 and less RNA2 than that of wt in both P1 viruses and P2 viruses (Fig. 5h). This is consistent with the reduced cellular RNA2 yield associated with HD2 (Fig. 5e). In mutant viruses, both HD1 and HD2 showed a significantly increased fraction of host RNA encapsidation in both P1 and P2 viruses (Fig. 5i). Taken together, this suggests the heterodimer stem plays a role in ensuring FHV genome packaging specificity.

Several additional mutants were also designed (Fig. S8) to confirm whether the observed phenotypes are indeed related to the disruption of heterodimer in HD1 and HD2. An alternative mutant (HDalt) was designed to retain the predicted base pairing of the heterodimer stem but with alternative synonymous codons. HDalt featured four mutations (two on each strand), which fully exhausted available synonymous codons. A swapping mutant (HDswap) swapped the predicted RNA1 sequence of the heterodimer stem with the reverse complementary RNA2 sequence. This retained the predicted base pairs but replaced amino acid residues. Two recovery mutants (HD1rec and HD2rec) were designed to restore the disrupted base pairs in HD1 and HD2. In HD1rec, four mutations were introduced to RNA2 to compensate the disrupted bases in HD1 while recoding three amino acid residues. In HD2rec, three and two mutations were introduced to RNA1 and RNA2, respectively, to compensate the mutations in the opposing strands. This recovered the disrupted bases in HD2 but recoded two amino acid residues in the open reading frames of each respective RNA. These additional mutants were created (primers listed in Table S2), verified by Sanger sequencing, and characterized for viability with methods stated above.

When measuring the relative cytotoxicity (Fig. S9a) with transfected plasmids, HDalt resulted in a slightly reduced (but not statistically significant) cytotoxicity compared to that of wt. In contrast, HDswap, HD1rec, and HD2rec were indistinguishable from the mock control (pMT-eGFP). From infected P1 cells, only HDalt produced detectable amounts of virus particles after sucrose gradient purification (Fig. S9b), whose yield was comparable to that of wt. The virus fraction from sucrose gradient-purified samples was analyzed with SDS-PAGE (Fig. S9c), which showed that capsids can be detected from only wt and HDalt. To investigate whether other mutants may produce marginal amount of virion that fell below the recovery threshold of sucrose gradient purification, the infected P1 cells were PEG purified, molecular weight filtered, and analyzed with SDS-PAGE (Fig. S9d). Only wt and HDalt showed clear bands corresponding to virus capsid. To understand whether other mutants may produce capsids but fail to assemble into mature virions, the cellular fraction from infected P1 cells was collected and analyzed with western blot (Fig. S9e). Again, capsids can only be observed from wt and HDalt. These observations are largely expected since HDalt is the only mutant that does not encode changes to the amino acid sequence. We further heterodimerized purified HDalt and analyzed the genomic RNA with non-denaturing agarose gel electrophoresis (Fig. S9f). HDalt formed heterodimer with the identical mobility shift pattern to that of wt. Importantly, we did not observe the heterodimer conformational change as that was observed for HD1 or HD2 (Fig. 4c). Overall, in spite of synonymously replacing several nucleotides, HDalt exhibited an identical phenotype to that of wt, suggesting that the previously observed deficiency in viability of the HD1 and HD2 mutants is indeed related to the disruption of heterodimer stem base pairing.

Following the recovery and the near wt behavior of HDalt, we also explored an alternative “cross-match” transfection strategy: one RNA component of HDalt was coupled with the other RNA component of wt virus (i.e., HDalt RNA1 + wtRNA2; or wtRNA1 + HDaltRNA2) (Fig. S9). This is to create base-pairing mismatches in heterodimer region, without protein-coding changes, to determine whether the wild-type activity of HDalt is dependent on maintenance of base pairing in the heterodimer stem. Such cross-match mutant viruses (namely: Alt1wt2 and Alt2wt1) resulted in comparable cytotoxicity (Fig. S9a) and virion yield (Fig. S9b) to that of wt and HDalt. No significant differences were found among wt, HDalt, Alt1wt2, and Alt2wt1. After heterodimerization, the encapsidated RNAs of Alt1wt2 and Alt2wt1 showed the same migration pattern to that of wt and HDalt particles (Fig. S9f), which is clearly different from the faster migration pattern of HD1 and HD2 (Fig. 4c). The cross-matched mutant viruses did not result in any significant disruption of heterodimer or measurable viral properties. The disruption of base paring in Alt1wt2 and Alt2wt1 was limited (Fig. S9): in Alt1wt2, the U and G mutations in HDalt RNA1, respectively, resulted in one and two hydrogen bond losses from the wt C-G and U-A pairings for a total of three H bond losses; while in Alt2wt1, the C and C mutations in HDalt RNA2, respectively, resulted in one H bond gain and two H bond losses from the wt G-U and U-A pairings, for a total of one H bond loss. Such changes are likely to be insufficient to disrupt the long double-stranded stem in the heterodimer. Therefore, these changes are likely to be even less significant to trigger any potential global RNA rearrangement as seen from HD1 and HD2 (Fig. 4), which may be the culprit of the observed packaging and thermostability defects.

### Disrupted heterodimer reduced virion thermostability

**Fig 6:**
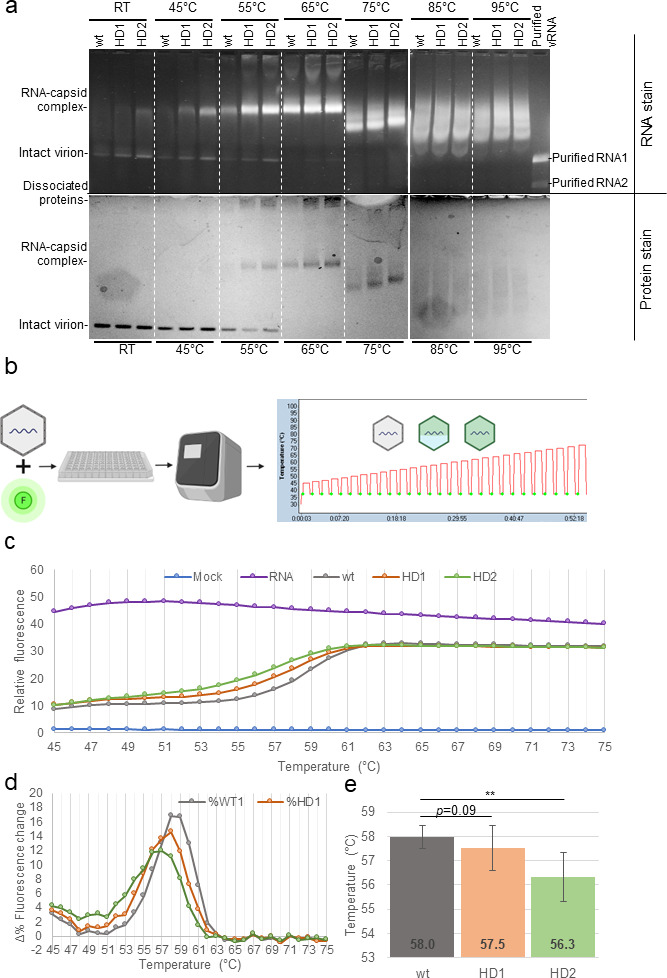
HD1 and HD2 mutants exhibit reduced virion thermostability. (**a**) Wt, HD1, and HD2 particles were heated for 15 min at the temperatures indicated and loaded onto a non-denaturing agarose gel containing nucleic acid stain, which was subsequently post-stained with Coomassie blue to reveal protein content. HD1 and HD2 constantly showed greater permeability of nucleic acid stain up to 75°C. At 55°C, HD1 and HD2 showed substantially reduced amount of intact virion and increased amount of RNA-capsid complex. At 75°C, RNA-capsid complex of three particles showed different mobility shift patterns, indicating further dissociation between protein and RNA. No difference among wt, HD1, and HD2 particles was observed above 85°C. (**b**) A high-throughput fluorimetry assay was designed to monitor thermostability changes of virus particles. Purified mutant or wt virions were mixed with a fluorescent nucleic acid dye and heated for 1 min per Δ1°C, ranging from 45°C to 95°C. Each heating cycle was followed by a rapid cooling to 37°C and a brief incubation (10 s) at 37°C. Green dots represent measurement of relative fluorescence, which is at the end of each colling cycle at 37°C. (**c**) In comparison to wtFHV, both HD1 and HD2 showed increased relative fluorescence at lower temperatures. (**d**) Δ%fluorescence/°C demonstrated that with wtFHV, fluorescence increase started at 54°C and peaked at 60°C. HD1: fluorescence increase started at 52°C and peaked at 59°C.HD2: fluorescence increase started at 49°C and peaked at 57°C. (**e**) Temperature required to reach 50% of relative fluorescence increase. (**c–e**): One-tailed Student *t*-test for means (equal variances), with α = 0.05, *N* = 12 (two biological replicates × six technical repeats).

We next sought to understand whether disruption of the identified heterodimer stem could interfere with virion thermostability. We designed a molecular assay (Fig. 6a), in which 50 µg of the quintuple-purified virions was heated at various temperatures (from 45°C to 95°C) for 15 min and subsequently loaded to a 1% non-denaturing agarose gel pre-stained with nucleic acid dye. After imaging, the same gel was post-stained with Coomassie blue to assay protein content. We expected that intact virions would be less permeable to nucleic acid dye and preclude fluorescent staining, whereas heat-treated particles would lose their structural integrity and thus be permeable to the intercalating dye resulting in an increase in measured fluorescence. We observed that both HD mutants were more permeable than wt to nucleic acid stain even at RT, which is characterized by brighter fluorescence of the RNA-capsid complex band(s). Such permeability difference was consistent when heating particles up to 75°C. The most substantial difference could be observed at 55°C, at which temperature both HD1 and HD2 exhibited higher nucleic acid dye permeability, greater amount of RNA-capsid complex, greater amount of fully dissociated proteins (protein-stained gel), and fewer intact virus particles (protein-stained gel). All particles appeared to lose integrity at 65°C; at which point, fluorescence peaked. However, at 75°C, the RNA-capsid complexes of wt, HD1, and HD2 showed different electrophoretic mobility shift patterns, indicating different degrees of protein-nucleic acid separation. Above 85°C, all viruses further disintegrated, and no difference was distinguishable between mutant(s) and wt. Despite the high temperature, there was still evidence for retained RNA-capsid interactions as multiple RNA species were observed to migrate slower than purified viral RNA1 or RNA2. It has been previously demonstrated that FHV virion comprises extensive RNA-capsid interactions (26) that may contribute to the heat resistance observed in this study.

To test virion thermostability in a high-throughput manner, we developed a fluorometric method on a qRT-PCR machine to detect fluorescence increase during particle disassembly upon temperature increases (Fig. 6b). Similar to the gel electrophoretic approach above, this measures fluorescence change after the heat-induced permeability of virus particles to intercalating nucleic acid dyes. Following each heating step (1 min), relative fluorescence was measured after a rapid cooling to 37°C (10 s) to minimize temperature-dependent effects on dye fluorescence absorbance. With this method, we monitored the thermostability changes of wt, HD1, and HD2. For all three particles, the fluorescence increase displayed a sigmoidal curve (Fig. 6c), which was characterized by a rapid increase in fluorescence at approximately 51°C and a plateau of fluorescence above 61°C (Fig. 6c and d). For wt virions, the greatest rate of fluorescence change was observed at 58°C–59°C, indicating the temperature that leads to greatest disassembly of the virus particle and exposure of genomic content to the intercalating dye (Fig. 6d). This is consistent with our native gel electrophoresis assay that also indicated particle disassembly at between 55°C and 65°C (Fig. 6a). Interestingly, both HD1 and HD2 showed an increase in fluorescence at lower temperatures than that of wt (Fig. 6c and d) and consistently showed more fluorescence than that of wt between 45°C and 63°C. Above 63°C, the relative fluorescence of all three particles plateaued and were equal. This also confirms equimolar pooling of particles in our experimental setup. By comparing the Δ rates of particle disassembly (defined here as the Δ percentage of relative fluorescence change, Fig. 6d), HD1 exhibited the greatest rate of fluorescence increase at 58°C, which rapidly drops thereafter. HD2 showed the greatest rate of fluorescence increase at 57°C. The melting temperature of a particle (Tm) is defined in this study as the temperature required to reach 50% fluorescence increase of the particle (Fig. 6e). On average, both HD1 and HD2 showed lower Tm(s) than that of wt. HD2 showed a significantly reduced Tm of 56.3°C, suggesting that the disrupted heterodimer stem in HD2 significantly compromised the thermostability of the virion.

Altogether, these results demonstrate differences in thermostability between wt and the mutant HD1 or HD2 particles as the result only of synonymous changes in the genomic RNA sequences that disrupt RNA higher-order structure inside the virus particle in the absence of any change in the proteinaceous virus capsid shell itself.

## DISCUSSION

### FHV heterodimer and biological importance

Prior to this study, a biological role for the Nodavirus genome heterodimer had only limited evidential support (10), including: (i) that the formation of RNA heterodimer is conserved among different viruses of the Nodavirus family, such as Nodamura virus and Flock House virus and (ii) that the formation of FHV RNA heterodimer necessitated virion structure. The latter point is in clear contrast to the genome homo-dimerization of retroviruses, in which two identical genomic RNA can dimerize *in vitro* spontaneously (57).

The Nodavirus heterodimers were generated after heating the virions to 65°C. Although it is unlikely that any intracellular compartment can reach such temperatures under physiological conditions, this heating can be considered as an accelerated but analogous representation of the dynamic energy exchanges between virus particle and cellular microenvironments. For example, it has been demonstrated that when heated to 56°C *in vitro*, the conformational change of poliovirus fully recapitulates the same *in vivo* receptor-induced viral uncoating process (16). For FHV and other Nodaviruses, we hypothesized that the heating-cooling cycle mimics and expediates unknown dynamic interactions between virions and intracellular components, which significantly alter the energy barrier of conformational changes *in vivo* and in virion. Since heterodimer formation requires virion structure (57), we also speculate that the heterodimer formation necessitates the specific architecture of virus capsid shell, which may spatially confine the free movement of RNA1 and RNA2 in a specific manner in the virion. The heating-cooling cycle (and the represented conformational changes) rearranged specific hydrogen bonds at these confined positions, allowing heterodimerization to occur.

In this study, we provide additional evidence to support a biological role for the FHV heterodimer. We found that: (i) heterodimer is formed as a result of RNA1-RNA2 intermolecular base pairing and can be crosslinked with AMT (Fig. 1b); (ii) the heterodimer formation is conserved in defective virus particles despite large internal deletions in the defective genomes (Fig. 3); (iii) the disruption of one heterodimer stem resulted in significant conformational change of the heterodimer (Fig. 4c); (iv) such disruption further attenuated virion assembly (Fig. 5a through d), RNA replication (Fig. 5e through g), genome packaging specificity (Fig. 5h and i) as well as the thermostability of virion (Fig. 6). Altogether, these observations demonstrate that the intermolecular base pairing of genomic RNAs is essential to the virus lifecycle and that disrupting such interactions impacts virus fitness.

To study and observe the heterodimer, we found that the specific RNA extraction method and conditions were critical. Following the heating and slow cooling of virions, a number of commercially available RNA extraction/purification kits/methods were tested. In this study, heterodimer could only be recovered with two methods: (i) proteinase K digestion followed by solid-phase reversible immobilization bead pull-down and ethanol wash (reduced yield) and (ii) a silica-based RNA purification kit (Quick-RNA Viral Kit, Zymo Research), which uses sodium iodide as the leading chaotic reagent (used throughout this study). Interestingly, other silica-based RNA extraction kits (e.g., RNA Clean & Concentrator and Direct-zol RNA prep from Zymo Research) or Trizol-based (Invitrogen) phase separation methods failed to maintain the RNA1-RNA2 association.

It is conceivable that the FHV genome consists of multiple heterodimer sites beyond those identified in this study. The disruption (Fig. 4c) or RNase H digestion (Fig. S6) on both sides of a single heterodimer site was demonstrated to be insufficient to fully dissociate RNA molecules. The existence of numerous heterodimer sites and the extensive RNA1-RNA2 interactions are also supported by previous findings that the majority of packaged FHV RNAs were in double-stranded conformation (26) and that the FHV genome forms a highly ordered dodecahedral RNA cage inside virion (25).

We designed synonymous mutations to disrupt the predicted base pairing at the heterodimer stem (RNA1: 1094–1102; RNA2:277–285). Such disruption was determined to have little impact upon the replication of viral RNAs and subsequent expression of viral capsid protein but rather had a profound effect upon virion assembly and genomic encapsidation. The connection between viral genome dimerization and packaging has been previously described for retroviruses such as in human immunodeficiency virus 1 (58–60) and murine leukemia virus (61, 62), as well as for multi-segmented dsRNA orbiviruses such as bluetongue virus (63) and rotavirus (64). For FHV and other alphanodaviruses, we speculate that the RNA1-RNA2 heterodimerization contributes to virus packaging efficiency via non-covalent coupling of each RNA that subsequently ensures stoichiometric RNA encapsidation.

One unexpected finding is that the HD2 mutant produced significantly more sgRNA3 than wt FHV. It has previously been demonstrated that two *cis*-acting elements on RNA1 (nt. 1229–1239 and nt. 2282–2777) regulate the production of sgRNA3 (18), whose replication further *trans*-activates the replication of RNA2 (52, 56). The RNA3-dependent replication of RNA2 reciprocally suppresses RNA3 synthesis via an unknown mechanism (55, 56). In this study, the discovered heterodimer stem is not adjacent to any known *cis-* or *trans-*acting elements. Nonetheless, the HD2 mutant exhibited increased sgRNA3 and reduced RNA2 replication. This suggests that the region forming the heterodimer stem (RNA1: 1094–1102, RNA2: 277–285) may also facilitate the RNA3-RNA2 *trans*-activation or regulation of RNA3 expression and may have further roles in maintaining intracellular RNA1-RNA2 stoichiometry.

It is interesting that, although HD1 was designed to contain more disruptive mutations to the heterodimer interaction site, HD2 exhibited more disruptive phenotypes, such as decreased virion yield (Fig. 5c), perturbed RNA1:RNA2 encapsidation ratios (Fig. 5h), and reduced thermostability (Fig. 6e). This suggests that the genotypic introduction of single-stranded bases does not guarantee base-pair disruptions in the virus, as the introduced single-stranded RNA bases can be base paired elsewhere. This is also supported by our DMS-MaPseq results (Fig. 4b).

Based on the defective phenotypes of HD1 and HD2, we sought to create additional viral mutants to attempt to recover the disrupted heterodimer and, thereafter, restore wild-type viral properties. HDswap, HD1rec, and HD2rec all contained non-synonymous mutations in heterodimer region and were unable to produce viable viruses (Fig. S8 and S10). An alternative mutation (HDalt) contained four alternative mutations to preserve base pairing and maintained protein coding while disrupting as far as possible in this region to test whether unexpected sequence-dependent factors might be at play (such as unknown host RNA-binding proteins). HDalt exhibited near wild-type behavior (Fig. S8 and S10), providing some evidence against a critical role of unknown RNA motif or binding site. Two cross-matched mutations were also explored (namely Alt1wt2 and Alt2wt1), which also delivered wt-like phenotypes (Fig. S9 and S10), in spite of minor disruption in the heterodimer stem. These attempts are in favor of the hypothesis that heterodimer disruption led to the defective phenotypes of HD1 and HD2. However, we cannot fully rule out the possibility that other unknown and additional factors beyond disruption of the heterodimer stem may also contribute to the packaging and thermostability deficiency of HD1 and HD2 mutants.

### XL-ClickSeq and dsRNA probing

NGS-coupled RNA structure probing methods such as SHAPE-MaP (65) and DMS-MaPseq (66) have enabled high-throughput RNA secondary structure mapping on genomic and transcriptomic scales, in both *in vitro* and *in vivo* settings. Their correspondent chemicals [SHAPE reagents such as 1-methyl-7-nitro-isatoic anhydride (1M7) and dimethyl sulfide] effectively methylate single-stranded RNA bases that can subsequently be detected as single-nucleotide mutations in NGS (67, 68). As such, double-stranded RNA is typically inferred reciprocally, with the support of computational thermodynamic prediction algorithms (26). Psoralen-based crosslinking methods thus provide an alternative and direct approach to specifically probe dsRNAs. Here, we developed a novel method (XL-ClickSeq) as an alternative to ssRNA chemical probing methods (such as SHAPE-MaP and DMS-MaPseq) and dsRNA crosslinking and proximal ligation methods (69, 70). XL-ClickSeq specifically probes crosslinked dsRNA regions with a highly efficient protocol that only requires a single crosslinking step prior to routine NGS library constructions using “ClickSeq.” XL-ClickSeq reveals dsRNA sites by marking the approximate location of the predicted crosslinks, therefore providing a near-nucleotide resolution of the covalent bond(s) that resides in dsRNAs (we estimate within 10 nts. of the XL-ClickSeq site). Of note, XL-ClickSeq does not provide the sequence of the crosslinked counterpart. This is in contrast to approaches such as PARIS (69) or COMRADES (70) that sequences both strands of a crosslinked dsRNA after proximity ligation, allowing for identification of long-distance and intermolecule RNA-RNA interactions. In this study, we used thermodynamic prediction to discover potential long-distance RNA-RNA interactions indicated by XL-ClickSeq. Although such an approach is limited in throughput, we think XL-ClickSeq provides a simple and convenient solution to address whether an RNA site is base paired or not, which is the most suitable for proof-of-principle research such as this study to understand and determine the biological impact of viral genomic heterodimerization.

Although it would be ideal to conduct the AMT-based crosslink with the least amount of disruption of virus particle (such as within intact virions), the rigid capsid structure (which is impermeable to nucleic acid stain when intact) prevents the penetration of psoralen derivatives. AMT crosslinking has been attempted with intact virions, virions of different pH, salt, buffering conditions, and virions in EGTA (71), with no observation of crosslinking to encapsidated viral RNAs. Performing crosslinking within infected cells prior to virus packaging is also likely to be problematic since the FHV genome packaging process is highly dynamic and requires delicate coordination between RNA and capsid to allow the compression of RNA genome into the confined virion structure (26) and since there may well be genomic RNA rearrangements within assembled virus particles during the process of virus maturation that occurs after cell egress.

### High throughput virion thermostability assay

In this study, we presented a high-throughput method to track the change in permeability of virions to intercalating nucleic acid dyes during thermo-denaturation. The approach requires a fluorescent nucleic acid stain with affinity to both ss- and ds-RNA (such as “GelStar” in this study) and can be conducted in any fluorescence-based qRT-PCR machine with correspondent emission/excitation filters. As such, this approach could be easily applied in wide range of viruses. There are two essential pre-requisites to ensure the accurate interpretation of virus thermostability: (i) purity of viruses must be maximized as any co-purified or non-encapsidated nucleic acid can yield a high background fluorescence. In this study, each virus particle was subjected to five rounds of purification (1× PEG precipitation, 2× sucrose gradient ultracentrifugation, and 2× molecular weight filtration) as well as two rounds of RNase/DNase digestion (ii). After each heating step, it is important to measure fluorescence at a constant temperature (37°C in this study) to avoid temperature-induced absorbance changes of the nucleic acid stain. With this simple high-throughput method, we discovered that the mutant viruses with a disrupted heterodimer stem exhibited reduced thermostability (Fig. 6b). This is consistent with findings from molecular assays (Fig. 6a).

It has been speculated that FHV RNA secondary structures may serve as scaffold to maintain structural stability (72). We have also previously demonstrated using NGS-based RNA-protein crosslinking approaches that mature FHV virions contain extensive RNA-capsid interaction sites and that these sites are enriched in double-stranded regions of the RNA genome (26). The reduced thermostability of FHV mutants demonstrates that disruption of RNA secondary structure of encapsidated viral genome has a direct impact on the virus supramolecular structure and particle thermostability. As there are likely multiple heterodimer sites in the encapsidated RNAs, it is foreseeable that these RNA1-RNA2 interactions collectively contribute to the overall structure stability and physical integrity of FHV virions.

## Data Availability

The raw sequencing data of this study are available in the NCBI sequence read archive (SRA) with accession number: PRJNA858427.

## SUPPLEMENTAL MATERIAL

Data Set S1 jvi.01820-23-s0001.xlsx XL-clickseq revealed coverage changes and moving fold changes.

Supplemental figures jvi.01820-23-s0002.pdf Figures S1 to S10.

Supplemental tables jvi.01820-23-s0003.docx Tables S1 and S2.

ASM does not own the copyrights to Supplemental Material that may be linked to, or accessed through, an article. The authors have granted ASM a non-exclusive, world-wide license to publish the Supplemental Material files. Please contact the corresponding author directly for reuse.
