# Supplementary material for: Bipartite viral RNA genome heterodimerization influences genome packaging and virion thermostability
Source: J Virol. 2024 Feb 8;98(3):e01820-23. doi: 10.1128/jvi.01820-23 (PMC10949487; doi:10.1128/jvi.01820-23)
Supplement: Supplemental tables — Tables S1 and S2. [file jvi.01820-23-s0003.docx]

| **Supplemental Table 1. Primers used for HD mutants** | | | |
| --- | --- | --- | --- |
| HD1 | RNA1 | Forward primer | AGAGCGTGAACGCTAGGCTTATCGGTATGGGA |
|  |  | Reverse primer | TAGCGTTCACGCTCTGGGTGGCGGATAATC |
|  | RNA2 | Forward primer | AGATAGATTTGAAGGCAAAGTGGTCAGCCGAAAG |
|  |  | Reverse primer | CCTTCAAATCTATCTGGTATTCCCTTACCGGGGT |
| HD2 | RNA1 | Forward primer | AGTCTGTGAACGCTAGGCTTATCGGTATGGGA |
|  |  | Reverse primer | TAGCGTTCACAGACTGGGTGGCGGATAATC |
|  | RNA2 | Forward primer | AGATCGTTTTGAAGGCAAAGTGGTCAGCCGA |
|  |  | Reverse primer | CCTTCAAAACGATCTGGTATTCCCTTACCGGGGTC |

**Supplemental Table S1. Primers designed for In-Fusion Cloning (TaKaRa) to create mutants HD1 and HD2.** Red bases represents mutated nucleotides

| **Supplemental Table 2. Primers used for additional mutants** | | | |
| --- | --- | --- | --- |
| HDalt | RNA1 | Forward primer | AATCGGTTAACGCTAGGCTTATCGGTA |
|  |  | Reverse primer | TAGCGTTAACCGATTGGGTGGCGGA |
|  | RNA2 | Forward primer | TGACCGATTTGAAGGCAAAGTGGTCA |
|  |  | Reverse primer | CCTTCAAATCGGTCAGGTATTCCCTTAC |
| HDswap | RNA1 | Forward primer | TGATAGATTACGCTAGGCTTATCGGTAT |
|  |  | Reverse primer | TAGCGTAATCTATCAGGGTGGCGGATAATCCCGAG |
|  | RNA2 | Forward primer | AATCTGTCATGAAGGCAAAGTGGTCAG |
|  |  | Reverse primer | CCTTCATGACAGATTGGTATTCCCTTACCGGGGTC |
| HD1rec | RNA1 | HD1 RNA1 plasmid unchanged | |
| HD1rec | RNA2 | Forward primer | ACCTCATGGTCTTGAAGGCAAAGTGGTCAG |
|  |  | Reverse primer | TCAAGACCATGAGGTATTCCCTTACCGGGGT |
| HD2rec | RNA1 | Forward primer | CCCAGACGGTGTACGCTAGGCTTATCGGT |
|  |  | Reverse primer | CGTACACCGTCTGGGTGGCGGATAATCCCGA |
|  | RNA2 | Forward primer | ACCACATCGTCTTGAAGGCAAAGTGGTCAG |
|  |  | Reverse primer | TCAAGACGATGTGGTATTCCCTTACCGGGGT |

**Supplemental Table S2. Primers designed for In-Fusion Cloning (TaKaRa) to create alternative or recovery mutants.**
